# Text-Guided Feature Completion in the CLIP Embedding Space for Occlusion-Robust Facial Expression Recognition

**DOI:** 10.3390/s26206551

**Published:** 2026-10-15

**Authors:** Jeonguk Seo, Minsam Ko

**Affiliations:** Department of Applied Artificial Intelligence, Hanyang University, 55, Hanyangdaehak-ro, Ansan-si 15588, Republic of Korea; sju02051@hanyang.ac.kr

**Keywords:** facial expression recognition, occlusion robustness, vision–language models, CLIP, feature completion, self-supervised reconstruction, affective computing

## Abstract

Camera-based affective sensing degrades sharply when the face is partially occluded by hands, masks, eyeglasses or headwear. Existing occlusion-robust facial expression recognition (FER) methods either discard the information carried by occluded regions through attention re-weighting, hallucinate it back at the pixel level, or restore it in a purely visual latent space; none of them can consult an external semantic description of what the hidden region ought to look like. We propose *TeCoRe*, a text-conditioned completion-and-recognition framework that restores occluded patch tokens directly inside the joint vision–language embedding space of a frozen CLIP encoder. A lightweight visibility gate estimates a per-patch occlusion probability; a two-layer transformer decoder reconstructs the occluded tokens using class-level textual emotion embeddings, produced by the frozen CLIP text encoder, as both cross-attention memory and an alignment target; and a gated blend passes clean tokens through unchanged so that completion cannot corrupt visible evidence. TeCoRe is trained as supervised FER using the ground-truth emotion labels for classification and text alignment, together with an auxiliary self-supervised reconstruction objective. Because the occlusion masks are synthesised during training, the pre-occlusion CLIP tokens of the same image provide the reconstruction target without requiring additional manual annotation. The CLIP backbone is completely frozen—no low-rank adapters, no landmarks, no segmentation priors—and only about 27 M head and decoder parameters are trained. On the standard RAN occlusion and pose subsets, TeCoRe reaches 91.42% on Occlusion-RAF-DB, 84.79% on Occlusion-FERPlus, and 62.42% on Occlusion-AffectNet. It achieves the highest accuracy in our comparison on Occlusion-RAF-DB and on the RAF-DB and FERPlus pose subsets, while remaining competitive but not leading on the FERPlus and AffectNet occlusion subsets. The ablation and control studies are reported as three-seed means so that the small differences between variants are not read off a single initialisation. A backbone-generality control shows positive mean gains from the proposed modules across all three evaluated frozen CLIP configurations, with the largest gain observed in the main ViT-L/14@336 setting. These results indicate that performing occluded-token completion in a joint vision–language space, with class-level text embeddings providing shared conditioning to the completion process, is an effective and parameter-efficient route to occlusion-robust FER.

## 1. Introduction

Facial expressions are one of the densest non-verbal channels available to a vision sensor, and automatic facial expression recognition (FER) now underpins driver-state monitoring, tele-health, human–robot interaction and virtual-reality (VR) presence. Under laboratory conditions the problem is close to being solved. Under deployment conditions it is not: the moment a hand, a surgical mask, a pair of sunglasses or the rim of a head-mounted display covers part of the face, accuracy collapses. This is not an edge case but the normal operating regime of any camera placed in the wild, and it is the single largest gap between reported and delivered FER performance.

Three families of remedies dominate the literature. *Attention re-weighting* methods, beginning with Region Attention Networks, learn to down-weight the occluded regions. They are stable and cheap, but they are fundamentally subtractive: the information carried by the occluded patches is discarded rather than recovered, so the model is asked to classify from strictly less evidence. *Pixel inpainting* methods reconstruct the missing face image before recognition. They are additive, but they solve a harder problem than necessary—photo-realistic synthesis—and inherit its instability and hallucination risk. *Prior-injection* methods supply facial landmarks or semantic-segmentation maps as auxiliary guidance and fine-tune a heavy backbone end to end; they are effective but expensive, and they depend on the accuracy of the external prior. A recent example, ORSANet, combines segmentation and landmark priors and reports strong results, but it evaluates on its own occlusion split rather than on the standard RAN occlusion/pose subsets used throughout this paper; its numbers are therefore not directly comparable with those reported here, and we do not include it in our comparison tables.

A fourth and more recent direction restores the missing information in a latent space rather than in pixel space. Latent-OFER, for instance, detects, masks and reconstructs occluded regions using latent vectors. This is the right level of abstraction—one does not need to know the exact texture of a hidden mouth, only its expressive meaning—but the latent space in question is purely visual. Consequently the reconstruction has no external reference: the model must guess what belongs in the hole using only the surrounding pixels.

Our observation is that a vision–language model provides an additional source of class-level semantic conditioning. In the joint embedding space of CLIP, *image* representations and *textual* emotion descriptions are represented in a shared feature space. If completion is performed inside that space, the full bank of class-level emotion embeddings can be provided to the decoder as a shared semantic reference while it reconstructs occluded visual features. Importantly, the ground-truth emotion is not available at inference time, and the decoder is not given the embedding of the correct class alone. Rather, it receives the entire class-level text bank as conditioning memory. This form of vision–language conditioning is unavailable to purely visual feature-completion methods and motivates the design of TeCoRe.

Concretely, we propose **TeCoRe** (Text-Conditioned Completion and Recognition). A frozen CLIP ViT-L/14@336 encoder produces N=576 patch tokens. A small visibility gate predicts, per patch, whether it is occluded. A two-layer transformer decoder then reconstructs every patch token, using as cross-attention memory both the visibility-weighted image tokens and the projected class-level text embeddings. A gated blend keeps clean tokens untouched and substitutes reconstructed values only where the gate reports occlusion, which prevents the completion path from damaging visible evidence. TeCoRe is trained as supervised FER using the ground-truth emotion labels for classification and text alignment, together with an auxiliary self-supervised reconstruction objective. For this objective, the pre-occlusion CLIP tokens of the same image provide the reconstruction target without additional manual annotation because the occlusion is synthesised during training.

Two design decisions deserve emphasis. First, the backbone is *completely frozen*: no low-rank adapters inside CLIP, no landmark detector, no segmentation network. Only ≈27 M parameters are trained. Second, at test time neither masks nor clean views are required—the gate estimates occlusion, the decoder fills in, and the head classifies.

Our contributions are:

1. **Text-guided occluded-token completion in the CLIP embedding space.** To our knowledge we are the first to perform occluded-token completion for FER directly in the joint vision–language space. Completion is performed on features rather than pixels, with an auxiliary self-supervised reconstruction target derived from the corresponding pre-occlusion CLIP tokens, while frozen class-level textual emotion embeddings provide class-level conditioning to the completion process.
2. **A frozen-backbone, ≈27 M-trainable-parameter design** that uses no low-rank adaptation, no landmarks, no segmentation maps and no backbone fine-tuning, yet is competitive with or better than heavier fully-fine-tuned state-of-the-art models on the standard occlusion subsets.

## 2. Related Work

### 2.1. Occlusion-Robust FER

Real-world FER must contend with partial occlusion from hands, masks, eyeglasses and headwear, and with large head-pose variation, both of which remove or distort the facial regions that carry expressive information. Wang et al. established the now-standard evaluation protocol for this setting with Region Attention Networks (RANs) [1], which partition the face into regions and learn to re-weight them so that occluded or uninformative regions contribute less to the decision; the same work released the Occlusion- and Pose- subsets of RAF-DB, FERPlus and AffectNet that we adopt here. A large family of subsequent methods refines this idea of suppressing unreliable regions and emphasising reliable ones. EfficientFace [2] couples a lightweight convolutional extractor with channel–spatial modulators to capture both local and global cues; VTFF  [3] fuses two convolutional feature maps through visual-transformer self-attention; MRAN [4] models multi-level relationships between global and local features; and FG-AGR [5] and related attention-graph methods propagate information across facial regions. FERMixNet [6] takes an augmentation-centric route, synthesising training samples through a facial-mixing strategy that imitates real occlusion while preserving expression semantics.

A parallel line injects explicit structural priors. POSTER [7] and POSTER++ [8] use facial-landmark features to steer a cross-fusion transformer toward salient regions, and ORSANet [9] combines dense semantic-segmentation maps with sparse landmark geometry, fused through a multi-scale cross-interaction module and trained with a dynamic adversarial repulsion loss; ORSANet also contributes an occlusion-oriented dataset. These methods are effective but heavy: they fine-tune the full backbone and depend on auxiliary detectors—segmentation networks, landmark predictors—whose errors propagate into recognition. As noted in the introduction, ORSANet reports its results on a non-RAN occlusion split, so it is not directly comparable with the standard-protocol numbers we report.

What unites the attention- and prior-based families is that, with respect to the occluded region itself, they are subtractive: information behind the occluder is down-weighted or filtered, never restored. The model is therefore asked to classify from strictly less evidence than an unoccluded input would provide. Our method departs from this premise by attempting to restore the missing information rather than merely discount it.

### 2.2. Feature-Space Completion

If the goal is recognition rather than visualisation, restoring the occluded region in pixel space is unnecessarily hard: photo-realistic face inpainting is unstable and prone to hallucination, and any texture it invents is ultimately discarded by the classifier. This motivates restoring the missing content in a feature space instead. Latent-OFER [10] embodies this idea for occluded FER: it detects the occlusion, masks it, and reconstructs the occluded region using latent vectors before classification. Latent completion has likewise been explored elsewhere in vision, for instance in person re-identification and in masked-autoencoder-style representation learning.

All of these methods, however, reconstruct within a *unimodal visual* latent space. The only reference available to the reconstruction is the surrounding visual content, so the network must infer what belongs in the hole purely from the visible pixels of the same face. Our completion instead operates inside the *joint vision–language* embedding space of CLIP, where an image token and a textual emotion description are directly comparable. This is not a cosmetic change of backbone: it makes a textual emotion embedding a valid reconstruction reference. The decoder can be told, in the shared space, what the occluded region should mean—a form of guidance that is structurally unavailable to any method reconstructing in a purely visual space.

### 2.3. Vision–Language Models for FER

CLIP’s joint image–text space has been used for FER mainly to obtain semantic supervision from class-level text. CLIPER [11] attaches multiple textual descriptions per expression and trains in two stages to learn fine-grained, interpretable representations; CEPrompt [12] injects affective-concept prompts into the visual representation through a concept-guided adapter with knowledge distillation. Other recent work adapts the language side to each input, turning fixed class prompts into instance-aware text embeddings and aligning them with visual features as semantic priors.

These methods share a common structure: the text embedding functions as a classifier interface—a set of class prototypes that visual features are matched against, possibly after prompt adaptation. None of them performs *token-level reconstruction* of the visual representation. In our method the text embeddings play a different role entirely: they are cross-attention memory that conditions a completion decoder, and they are the alignment target that pulls the completed feature toward the correct emotion. The distinction is that we use text to guide the *reconstruction* of the visual representation, not merely to classify it.

## 3. Materials and Methods

### 3.1. Overview

**Figure 1:**
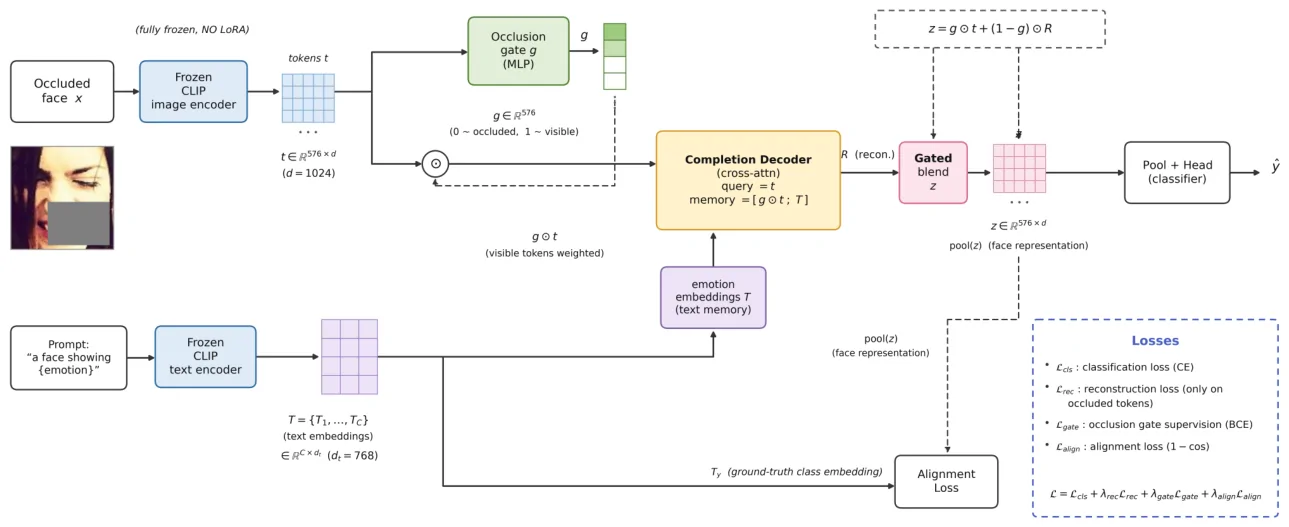
Overview of TeCoRe. The frozen CLIP image and text encoders (≈300 M parameters) are never updated; only the occlusion gate, completion decoder, projections and head (≈27 M parameters) are trained. Given an occluded face *x*, the image encoder produces patch tokens *t*; the occlusion gate *g* estimates per-patch visibility; the completion decoder reconstructs the tokens using the visibility-weighted tokens g⊙t together with the emotion embeddings *T* from the text encoder as cross-attention memory; and a gated blend z=g⊙t+(1−g)⊙R passes clean tokens through unchanged while substituting reconstructed values at occluded positions. The pooled representation is classified by a linear head. The clean view and the occlusion mask are used only during training; at inference neither is required.

Figure 1 shows the pipeline. An input image $x\in R^{3\times 336\times 336}$ is encoded by a frozen CLIP ViT-L/14@336 image encoder into a class token cls and N=576 patch tokens ${\left\{t_{i}\right\}}_{i=1}^{N}$, $t_{i}\in R^{D}$ with D=1024. A visibility gate scores each patch; a text-conditioned decoder reconstructs all patches; a gated blend selects between original and reconstructed tokens; and a linear head classifies. The CLIP text encoder, also frozen, provides class-level emotion embeddings $T\in R^{C\times J}$ with J=768.

### 3.2. Frozen Backbone and Patch Tokens

**Figure 2:**
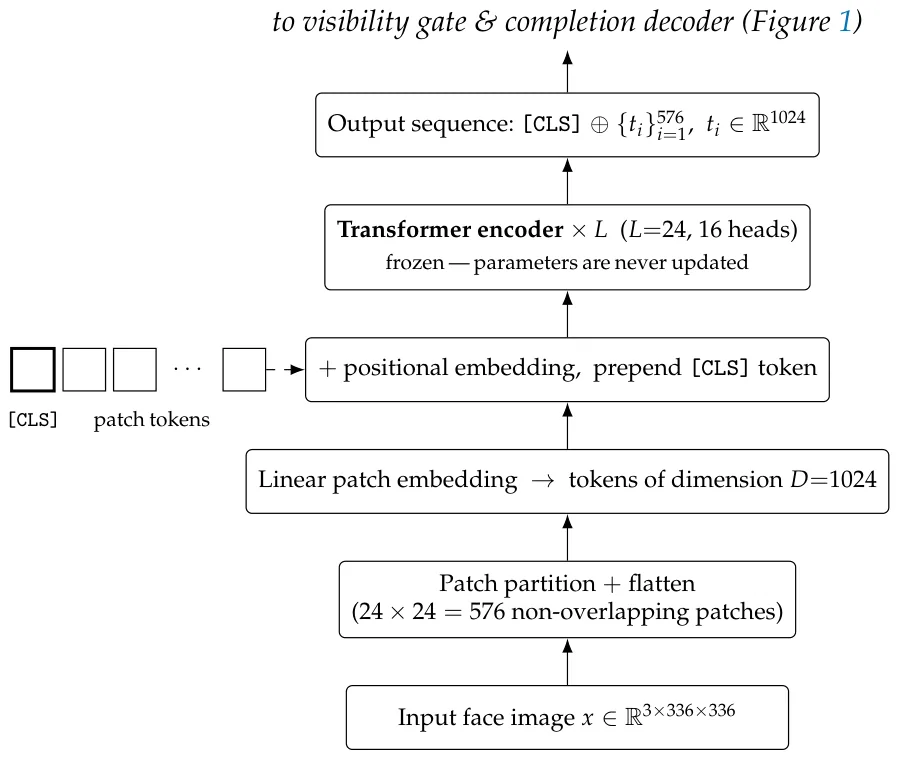
Architecture of the frozen CLIP ViT-L/14@336 image encoder that serves as the backbone. The input face is partitioned into 24×24=576 non-overlapping patches; each patch is linearly embedded to a 1024-dimensional token, a positional embedding is added, and a learnable [CLS] token is prepended. The token sequence is processed by L=24 pre-norm transformer encoder layers; a single layer is expanded on the right, showing multi-head self-attention and an MLP block, each wrapped by layer normalisation and a residual connection. The encoder is kept entirely frozen (≈304 M parameters, never updated). Its output—the [CLS] token together with the 576 patch tokens $\left\{t_{i}\right\}$—is handed to the visibility gate and text-conditioned completion decoder of Figure 1.

We use OpenAI CLIP [13] ViT-L/14@336 (openai/clip-vit-large-patch14-336). The 24×24 patch grid gives N=576 tokens of dimension D=1024; the joint projection dimension is J=768. All CLIP parameters are frozen throughout; no low-rank adapters are inserted. Figure 2 details the internal structure of this backbone. The input face is divided into 576 non-overlapping patches, each of which is linearly projected to a 1024-dimensional token; a positional embedding is added and a learnable [CLS] token is prepended, and the sequence is passed through L=24 pre-norm transformer encoder layers, each consisting of multi-head self-attention and a feed-forward block wrapped by layer normalisation and residual connections. Because the encoder is never updated, all of its representational capacity is inherited from large-scale vision–language pre-training and reused as-is; only the lightweight modules of Figure 1 that consume its output tokens are trained.

### 3.3. Visibility Gate

A per-patch visibility score is produced by a small MLP:

(1) $$g_{i}\;=\;\sigma \left(\mathrm{MLP}(t_{i})\right)\;\in \;\left[0,1\right],\;\mathrm{MLP}:\;\mathrm{LN}\;\to \;{\mathrm{Linear}}_{D\to 256}\;\to \;\mathrm{GELU}\;\to \;{\mathrm{Linear}}_{256\to 1},$$

where $g_{i}\;=\;1$ denotes a visible patch and $g_{i}\;=\;0$ a fully occluded one.

### 3.4. Textual Emotion Embeddings

Class-level prompts are encoded once by the frozen CLIP text encoder and kept fixed. For each class *c* we average the embeddings of three templates —“a photo of a {} face”, “a face showing a {} expression” and “a person who looks {}”—and $L_{2}$-normalise, yielding $T\in R^{C\times J}$.

### 3.5. Text-Conditioned Completion Decoder

A two-layer pre-norm transformer decoder (8 heads, dropout 0.1) takes the patch tokens as queries and, as memory, the concatenation of the visibility-weighted image tokens with the projected text embeddings:

(2) $$R\;=\;\mathrm{Decoder}\left(\underset{\mathrm{query}}{\underset{︸}{\left\{t_{i}\right\}}},\;\underset{\mathrm{memory}}{\underset{︸}{\left[\;g⊙t\;∥\;\psi_{t\to v}\left(T\right)\;\right]}}\right)\;\in \;R^{N\times D},$$

where ‖ denotes concatenation along the token axis and $\psi_{t\to v}\;:R^{J}\;\to \;R^{D}$ is a learned linear map. Each layer applies token–token self-attention, token–memory cross-attention and a feed-forward block.

### 3.6. Gated Blend and Classification

To prevent the completion path from corrupting visible evidence, reconstructed tokens replace the originals only in proportion to the estimated occlusion:

(3) $$z_{i}\;=\;g_{i}\;t_{i}\;+\;\left(1-g_{i}\right)\;R_{i}.$$

The classifier consumes the mean-pooled completed tokens concatenated with the CLIP class token:

(4) $$\bar{z}=\frac{1}{N}\sum_{i=1}^{N}z_{i},\;h=\left[\;\bar{z}\;∥\;\mathrm{cls}\;\right]\in R^{2D},\;\hat{y}={\mathrm{Linear}}_{2D\to C}\left(\mathrm{LN}(h)\right),$$

where $\bar{z}$ is the pooled token and *h* the classification feature. A second projection $\psi_{\mathrm{img}}\;:R^{D}\;\to \;R^{J}$, initialised from CLIP’s visual_projection weights, maps the pooled token into the joint space for text alignment:

(5) $$u\;=\;\frac{\psi_{\mathrm{img}}\left(\bar{z}\right)}{{∥\psi_{\mathrm{img}}\left(\bar{z}\right)∥}_{2}}\;\in \;R^{J}.$$

Trainable parameters total 27.06 M (27,060,488 for the 7-class RAF-DB configuration; the 8-class heads add fewer than 2100 parameters), of which the completion decoder accounts for 25.2 M and the gate, $\psi_{t\to v}$, $\psi_{\mathrm{img}}$ and head the remainder. At inference only the frozen CLIP vision encoder and its visual projection are executed, contributing 304.3 M (304,294,465) never-updated parameters, for a total inference-time footprint of ≈331 M. The frozen text encoder (123.7 M) is evaluated only once, before training, to pre-compute the fixed class-level emotion embeddings, and is not part of the inference graph.

### 3.7. Synthetic Occlusion and Dual-View Training

For every clean training image *x* we synthesise an occluded view $x^{\mathrm{occ}}$ and its patch-level mask $m\in {\left\{0,1\right\}}^{N}$, where $m_{i}\;=\;1$ marks an occluded patch. Synthetic occlusion consists of one or two random rectangles covering 10–50% of the image area, filled with noise, grey or black at random. Both views are passed through the frozen CLIP encoder; the clean view supplies the auxiliary self-supervised reconstruction target.

### 3.8. Training Objectives

Let $z_{i},R_{i},g_{i},\bar{z},u$ denote the quantities defined above on the *occluded* view, and let a ${\left(\cdot \right)}^{\mathrm{cl}}$ superscript denote the corresponding quantities on the *clean* view. We write cos(a,b) for cosine similarity and sg[·] for the stop-gradient operator.

Classification.

A label-smoothed (ε=0.1) cross-entropy is applied to both views:

(6) $$L_{\mathrm{cls}}\;=\;\mathrm{CE}\left({\hat{y}}^{\mathrm{occ}},y\right)\;+\;\mathrm{CE}\left({\hat{y}}^{\mathrm{cl}},y\right).$$

Reconstruction (self-supervised).

At each occluded position, the completed token $R_{i}$ is matched to the corresponding *pre-occlusion* CLIP token $t_{i}^{\mathrm{cl}}$ (used as a fixed target via stop-gradient):

(7) $$L_{\mathrm{rec}}\;=\;\frac{\sum_{i=1}^{N}m_{i}\;\left[\;1-\cos\left(R_{i},\;\mathrm{sg}\left[\;t_{i}^{\mathrm{cl}}\;\right]\right)\right]}{\sum_{i=1}^{N}m_{i}}.$$

Text-guided alignment.

The joint-space feature *u* is pulled toward the text embedding $T_{y}$ of the ground-truth emotion, on both views:

(8) $$L_{\mathrm{align}}\;=\;\left[\;1-\cos(u,T_{y})\;\right]\;+\;0.5\;\left[\;1-\cos(u^{\mathrm{cl}},T_{y})\;\right].$$

Gate supervision.

The visibility scores are supervised by the synthetic mask, with a weak prior that clean patches are fully visible:

(9) $$L_{\mathrm{gate}}\;=\;\mathrm{BCE}\left(g^{\mathrm{occ}},\;1-m\right)\;+\;0.1\;\mathrm{BCE}\left(g^{\mathrm{cl}},\;1\right).$$

Feature consistency.

The occluded-view pooled feature is drawn toward its clean-view counterpart:

(10) $$L_{\mathrm{cons}}\;=\;1-\cos\left({\bar{z}}^{\mathrm{occ}},\;\mathrm{sg}\left[\;{\bar{z}}^{\mathrm{cl}}\;\right]\right).$$

Total.

The full objective is a weighted sum,

(11) $$L\;=\;L_{\mathrm{cls}}\;+\;\lambda_{a}\;L_{\mathrm{align}}\;+\;\lambda_{r}\;L_{\mathrm{rec}}\;+\;\lambda_{g}\;L_{\mathrm{gate}}\;+\;\lambda_{c}\;L_{\mathrm{cons}},$$

with $\left(\lambda_{a},\lambda_{r},\lambda_{g},\lambda_{c}\right)=\left(0.5,\;1.0,\;0.5,\;0.5\right)$.

It is worth stating explicitly what is and is not supervised by text. The reconstruction ground truth is an *image* feature—the frozen CLIP vision encoder’s pre-occlusion patch token (1024-d). The text embeddings enter only (i) as cross-attention memory that conditions the decoder and (ii) as the alignment target of $L_{\mathrm{align}}$, where the ground-truth emotion label determines the corresponding class-level text embedding. Because we generate the occlusion, both the mask *m* and the clean reconstruction reference are obtained automatically and require no additional manual annotation beyond the emotion labels provided by the FER datasets.

### 3.9. Datasets and Evaluation Protocol

**Base datasets.** RAF-DB [14] (7 classes, 12,271 training/3068 test), FERPlus [15] (8 emotion classes assigned by majority vote over the crowd labels, after removing the *unknown* and *no-face* categories; 3573 test images), and AffectNet+ [16] (a soft-label re-release of AffectNet [17]; 8 classes, human labels 0–7, standard 4000-image validation set).

**Occlusion and pose subsets (RAN protocol).** Occlusion-RAF-DB (734), Occlusion-FERPlus (605) and Occlusion-AffectNet (683), together with Pose ≥ 30°/Pose ≥ 45° subsets of 1247/558 (RAF-DB), 1170/633 (FERPlus) and 1949/985 (AffectNet) images. For AffectNet, the RAN lists the original release by hash filename; we obtain the corresponding subsets from the redistributed AffectNet+ release by matching sorted numeric identifiers to the RAN validation order.

**Metrics.** Following the RAN protocol and subsequent occlusion-FER work, we report overall (top-1) accuracy on each subset.

### 3.10. Implementation Details

Optimisation uses AdamW with learning rate $10^{-3}$ for the trainable modules, weight decay $10^{-4}$, and a cosine schedule; there is no backbone learning rate because the backbone is frozen. We train for 25 epochs on RAF-DB and FERPlus, and 15 epochs on AffectNet with a class-balanced sampler drawing 40 k samples per epoch. Batch size is 64. Label smoothing is 0.1 and the loss weights are $\left(\lambda_{a},\lambda_{r},\lambda_{g},\lambda_{c}\right)=\left(0.5,\;1.0,\;0.5,\;0.5\right)$; these values, together with the learning rate and weight decay above, are the configuration selected on the held-out validation split in Section 3.11.

Each run is trained on a single NVIDIA RTX A6000 (48 GB) GPU (NVIDIA Corporation, Santa Clara, CA, USA) with PyTorch 2.5.1 (CUDA 12.1) under Python 3.10, using mixed-precision (bfloat16) training. Data augmentation on the clean view is deliberately minimal: resizing to the backbone resolution followed by a random horizontal flip, with no colour jitter, cropping or rotation; images are normalised with the standard CLIP mean and standard deviation. Synthetic occlusion is applied to the resized image before normalisation, and the clean and occluded views share the same geometric transform.

### 3.11. Data Splits, Seeds, Hyper-Parameter Search and Model Selection

**Splits.** From the RAF-DB training set (12,271 images) we draw a class-stratified 10% holdout of 1226 images and train on the remaining 11,045; FERPlus and AffectNet are split the same way. A synthetic occlusion of the validation split is generated once and stored, so the selection signal—*val-occ*, top-1 accuracy on the occluded validation split—is identical across every run, variant, backbone and seed in this paper. The official test sets (3068 images for RAF-DB) and the RAN occlusion/pose subsets derived from them are held out entirely.

**Seeds.** Three random seeds—42, 43 and 44—were fixed before any experiment reported here was run, and were never changed, re-drawn or added to. The same three are used for every configuration in this paper: the full model, every ablation variant, every backbone, every dataset, and every retrained baseline of Section 4.2. Every number we report is the mean over all three runs, with the standard deviation alongside it, so no value anywhere in this paper is selected from among seeds.

**Hyper-parameter search.** All hyper-parameters were selected on the held-out validation split using a single fixed seed, seed 42, with val-occ as the selection criterion. Starting from a centre configuration, one factor at a time was varied over $\eta \in \{3\cdot 10^{-4},5\cdot 10^{-4},10^{-3},2\cdot 10^{-3}\}$, weight decay $\in \{0,10^{-4},10^{-3},10^{-2}\}$, $\lambda_{a}\in \left\{0.25,0.5,1.0\right\}$, $\lambda_{r}\in \left\{0.5,1.0,2.0\right\}$, $\lambda_{g}\in \left\{0.25,0.5,1.0\right\}$, and $\lambda_{c}\in \left\{0.25,0.5,1.0\right\}$, together with three additional combinations of the individually best-performing factors. The final configuration used in all experiments was $\eta =10^{-3}$, weight decay $=10^{-4}$, and $\left(\lambda_{a},\lambda_{r},\lambda_{g},\lambda_{c}\right)=\left(0.5,\;1.0,\;0.5,\;0.5\right)$.

**Epoch selection.** We maintain an exponential moving average (EMA, decay 0.999) of the trainable weights, evaluate the EMA weights on val-occ after every epoch, and keep the epoch with the highest val-occ.

## 4. Results

### 4.1. Comparison with the State of the Art

Table 1 compares TeCoRe with published methods on the standard RAN occlusion and pose subsets. Every TeCoRe entry is the mean ± standard deviation over the three pre-fixed seeds of Section 3.11, each trained on a 90% split with its epoch selected on the held-out validation split and its test subset evaluated once. The competitor entries are the values reported in their own papers.

TeCoRe achieves 91.42% on Occlusion-RAF-DB and the best values on both RAF-DB pose subsets and both FERPlus pose subsets. On the FERPlus and AffectNet occlusion subsets, TeCoRe obtains competitive, but not leading, accuracy, scoring 1.2 percentage points below FERMixNet and 1.9 percentage points below AMP-Net, respectively.

The RAF-DB result is obtained with a frozen backbone and 27.06M trainable parameters, whereas every competitor in Table 1 fine-tunes its backbone end to end and AMP-Net additionally runs a face-landmark detector at inference. Because TeCoRe uses a larger backbone and a higher input resolution than most baselines, Table 1 alone cannot separate the contribution of the proposed modules from that of the backbone. Section 4.2 therefore provides a backbone-matched comparison with modern CLIP-based FER methods.

### 4.2. Comparison with CLIP-Based FER Under an Identical Protocol

CLIPER [11] and CEPrompt [12], introduced in Section 2.3, originally use CLIP ViT-B/16@224, whereas TeCoRe uses CLIP ViT-L/14@336. A direct comparison using the original configurations would therefore confound the effect of the proposed method with differences in backbone scale and input resolution, potentially favouring TeCoRe. To provide a fairer comparison under the same backbone setting, we replaced the original backbones of CLIPER and CEPrompt with the same CLIP ViT-L/14@336 backbone used by TeCoRe and retrained both methods from their released code.

All three methods were evaluated under the protocol of Section 3.11: the same train/validation split, the same stored validation occlusion, their respective published hyper-parameters, epoch selection on val-occ, the same three seeds, and a single evaluation of the test subset. We restrict the comparison to Occlusion-RAF-DB and report the trainable parameter count alongside accuracy in Table 2.

TeCoRe obtains 91.42% on Occlusion-RAF-DB, compared with 90.96% for CEPrompt and 90.64% for CLIPER, corresponding to differences of +0.45 and +0.77 percentage points, respectively. The main distinction is the number of trainable parameters. CEPrompt updates 277.9 M parameters and CLIPER 304.4 M, whereas TeCoRe updates 27.1 M and leaves CLIP frozen, corresponding to 10.3× and 11.2× fewer trainable parameters, respectively.

A sensing node serving *K* expression-related tasks stores 304.3+27.1K million parameters with TeCoRe against 304.4K million with CLIPER (413 M against 1.22 B at K=4; 108 MB against 1.22 GB per additional task). Re-specialising a deployed node for a new task therefore requires shipping only the task-specific head for TeCoRe rather than a new backbone.

TeCoRe is positioned for the setting in which a strong frozen vision–language encoder is available and shared across tasks.

### 4.3. Ablation Study

To quantify the contribution of each design choice, we ablate TeCoRe on Occlusion-RAF-DB. Every variant is trained with an identical protocol—frozen CLIP ViT-L/14@336, no low-rank adaptation, the hyper-parameters described in Section 3.11, 25 epochs, batch size 64, EMA, the epoch chosen on the held-out val-occ, and the test subset evaluated once—and differs from the full model in exactly one factor. Every entry is the mean ± standard deviation over the same three pre-fixed seeds.

Additive component ladder.

Table 3 switches on the two central mechanisms one at a time. A frozen CLIP encoder with only a linear head reaches 89.78%. Adding the gated completion decoder with an auxiliary self-supervised *image-feature* reconstruction objective, and no text at all, raises occlusion accuracy to 90.60% (+0.82). Turning on the text pathway—cross-attention memory together with the alignment loss—further raises occlusion accuracy to 91.42%, an increment of +0.82 over the text-free decoder. Relative to the frozen baseline, the complete system improves accuracy by +1.63 percentage points. The two successive increments are equal in magnitude at +0.82 points, indicating that both the completion decoder and the text pathway contribute to the overall improvement.

How the text is used.

We examine two complementary aspects of the text pathway: linguistic semantics (Table 4) and individual component contributions (Table 5). Panel (a) of Table 5 separates the two roles that the textual emotion embeddings play. Using them simultaneously as cross-attention memory *and* as the alignment target—the full model—gives 91.42%. Keeping only the alignment role reaches 91.33%, i.e., +0.73 over the text-free decoder and only 0.09 below the full model. Keeping only the memory role reaches 90.69%, i.e., +0.09 over the text-free decoder.

These results indicate that most of the observed gain from the text pathway is associated with the alignment loss, whereas the additional gain from using the text embeddings as cross-attention memory is small. The memory is retained in the architecture because it provides the text-conditioned input to the completion decoder. The following semantic-control experiment further examines the role of the textual guidance.

Does the text pathway require linguistic semantics?

The preceding ablation indicates that the benefit of the text pathway is primarily associated with the alignment objective. We next ask a complementary question: does this benefit depend on the linguistic meaning encoded in the class prompts? This distinction is important because the ground-truth emotion is unavailable at inference time. The decoder is therefore never given the embedding of the correct emotion class alone; instead, the entire bank of seven class-level text embeddings is supplied as cross-attention memory.

To test whether linguistic class semantics are necessary for the observed gain, we replace the prompt bank with seven learnable vectors initialized independently from N(0,1). This variant uses no text encoder, prompt templates, or other linguistic information. The vectors replace the class prompt embeddings in both the cross-attention memory and the alignment objective, and the model is retrained under the same protocol as the prompt-based model. Table 4 compares this control with the prompt-based model and the text-free completion model.

The prompt-based model achieves the highest mean accuracy at 91.42%, whereas replacing the prompt embeddings with learnable vectors containing no linguistic information yields 90.92%, a decrease of 0.50 percentage points. The learnable-vector control nevertheless remains 0.32 percentage points above the text-free completion model (90.60%). These results indicate that the benefit of the text pathway cannot be attributed solely to linguistic class semantics. At the same time, the higher mean accuracy of the prompt-based model is consistent with an additional benefit from the structured class-level text representations.

Accordingly, we do not interpret the class-level text bank as a *semantic oracle*. At inference time, it does not provide the target emotion or identify which class embedding is correct. Rather, the complete bank is available as a shared conditioning memory, and cross-attention can aggregate information from this set while completing the visual representation. We therefore do not assume that the decoder explicitly selects the correct emotion embedding from the bank. Together with the preceding component ablation, these results suggest that the text pathway’s improvement is primarily associated with the alignment objective, while the class-level text bank provides an additional conditioning signal whose benefit cannot be explained solely by its linguistic content.

Reconstruction target.

Panel (b) varies the target of the reconstruction loss $L_{\mathrm{rec}}$. The adopted target—the frozen CLIP *image* token—gives the highest mean accuracy at 91.42%. Pixel-space reconstruction reaches 91.14%, the frozen CLIP text embedding reaches 91.01%, and removing the reconstruction loss gives 90.64%.

The results show that the adopted frozen CLIP image-token target gives the highest mean accuracy among the evaluated settings, while removing the reconstruction loss produces the lowest mean accuracy. Pixel-space and CLIP text-embedding reconstruction give intermediate results. These results support the use of the adopted image-feature reconstruction target in TeCoRe.

For the pixel-space baseline, we kept the same two-layer completion decoder (d=1024, 8 heads) as the adopted model and added only a linear output head that maps each reconstructed token to the 588 RGB values of a 14×14 patch. The pixel prediction is used only for the reconstruction loss and is not fed to the classifier; classification still operates on the gated feature-space representation. The target is the patch-wise normalized RGB content of the clean image, and the reconstruction loss is MSE computed only over synthetically occluded patches, with the same loss weight ($\lambda_{r}=1.0$) and all other loss terms unchanged. Training uses exactly the same frozen CLIP ViT-L/14@336 backbone, data split, synthetic occlusion procedure, optimizer, learning rate, 25-epoch schedule, batch size, EMA-based epoch selection, and three seeds as the adopted model. Among the reconstruction settings evaluated here, the frozen CLIP image-token target achieved the highest mean accuracy; this comparison is limited to the specific pixel-reconstruction implementation described above and does not establish a general superiority of feature-space reconstruction over pixel-space reconstruction.

The gated blend is necessary.

Panel (c) ablates the soft visibility gate. The adopted soft-gated blend ($z_{i}=g_{i}t_{i}+\left(1-g_{i}\right)R_{i}$) reaches 91.42% on Occlusion-RAF-DB. Removing the gate entirely—so that the decoder overwrites *every* token, including visible ones—reduces accuracy to 90.19%, a decrease of 1.23 percentage points. Replacing the soft gate with a hard, thresholded gate further reduces accuracy to 89.74%, a decrease of 1.68 percentage points. Among the evaluated gating strategies, the soft visibility gate therefore gives the highest mean accuracy.

Taken together, the ablation results show that the gated completion decoder and the text pathway each increase the mean accuracy by 0.82 percentage points in the additive component ladder. Within the text pathway, the alignment-only variant remains close to the full configuration, whereas the memory-only variant remains close to the text-free decoder. The adopted frozen CLIP image-token reconstruction target gives the highest mean accuracy among the evaluated reconstruction targets, and the soft visibility gate gives the highest mean accuracy among the evaluated gating strategies.

### 4.4. Efficiency

TeCoRe trains only 27.06 M parameters (27,060,488) while keeping the frozen CLIP vision backbone (304.3 M) unchanged, so that of the ≈331 M inference-time parameters only about 8% are ever learned or stored per task. Its *training* and *storage* footprint is therefore small, and because the backbone is frozen a single encoder can be shared across tasks while only the lightweight completion head is specialised. We frame the efficiency contribution in these terms—trainable and storage cost. Table 6 breaks down where the parameters go: the completion decoder dominates the trainable budget, while the frozen CLIP encoder, though large, is fixed and shared and never enters the optimiser.

**Inference cost.** Measured with fvcore at 336 px, the full model requires ≈185 GFLOPs per image (multiply–accumulate count), of which the frozen CLIP encoder accounts for 175 GFLOPs and the trained completion head adds only 9.9 GFLOPs (5.3%). The proposed modules are therefore themselves lightweight; the inference compute is inherited from the ViT-L/14 backbone (a ResNet-18 baseline is ≈1.8 GFLOPs for comparison). Under mixed precision (bfloat16) on a single RTX A6000, TeCoRe runs at 16.6 ms/image at batch size 1 and 10.3 ms/image at batch size 32 (60–97 images/s), i.e., real time for a single camera stream. The efficiency claim should thus be read as low *trainable and storage* cost plus a lightweight added head, with inference computation set by the chosen backbone rather than by the proposed modules.

### 4.5. Qualitative Analysis

**Figure 3:**
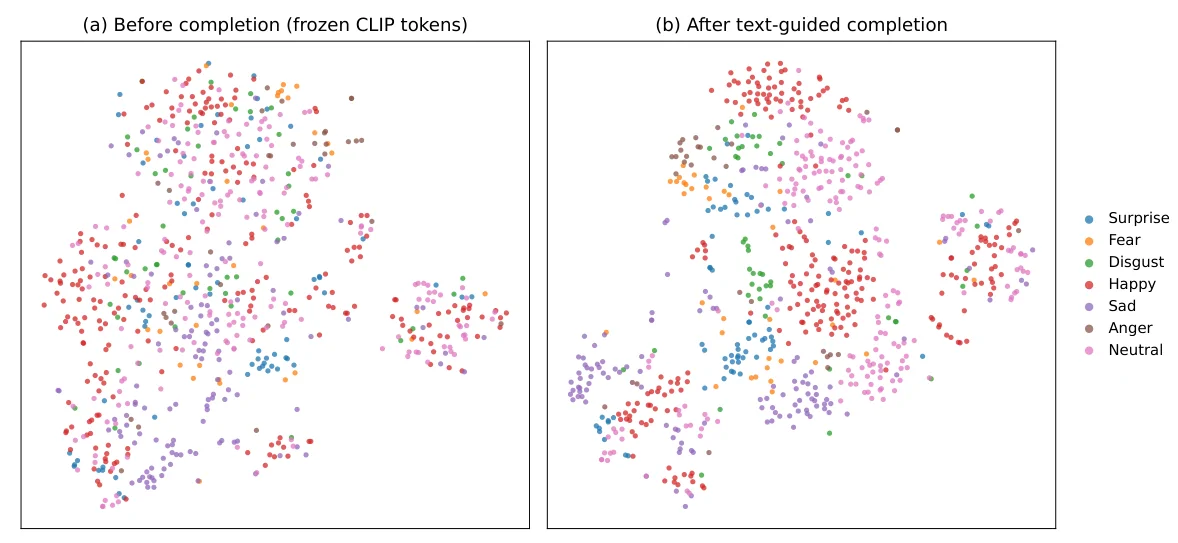
t-SNE of the pooled representation of the 734 Occlusion-RAF-DB test images, coloured by ground-truth emotion. Completion produces tighter, better-separated emotion clusters (10-NN probe accuracy 59.3%→80.3%).

Figure 3 visualises, with t-SNE, the pooled feature of every Occlusion-RAF-DB test image before and after text-guided completion. Before completion—the mean of the frozen CLIP patch tokens, which is what a frozen-backbone linear baseline classifies—the seven emotion classes overlap heavily, with *happy* and *neutral* in particular bleeding into one another. After completion the same images form markedly tighter and better-separated clusters: a 10-nearest-neighbour probe on the pooled feature improves from 59.3% to 80.3%. This 10-NN probe measures how separable the emotion classes are in the pooled feature space—a representation-quality diagnostic, not the end-to-end classification accuracy reported in Table 1. Because the two panels use identical inputs and differ only in whether the completion decoder is applied, the improved class structure is attributable to the text-guided completion rather than to the backbone.

### 4.6. Failure Analysis

The evaluation so far reports a single accuracy per subset, which says where the method stands but not where it breaks. We therefore partition Occlusion-RAF-DB by the kind of occlusion each image actually contains and report accuracy within each partition.

**Protocol.** Every one of the 734 images was assigned exactly one occluder type and exactly one occluded region, from the image alone, with no model output available at the time of assignment; the two partitions are orthogonal, so each image appears once in Table 7 and once in Table 8. The assignment was made after all training and model selections were complete and is used only to group results that were already fixed, so it cannot influence any reported number. We report accuracy for the adopted model (R2) and for the two reduced configurations of Section 4.3—R0, the frozen backbone with a linear head, and R1, which adds the completion decoder without text—over the same three seeds, so that each partition shows not only where the method fails but whether the proposed modules help there.

**Occluder type.** The gain over the frozen-backbone baseline is positive in nine of the twelve categories. The widest category, eyeglasses, gives +2.25 points over 193 images. The six categories above the rule in Table 7 contain at least 50 images and are therefore more informative than the smaller categories reported below the rule. One negative result is worth stating: on crop and out-of-frame images—where part of the face is missing from the frame rather than covered within it—the adopted model is 5.98 points *below* the linear baseline, the only category with more than ten images where this occurs. Completion appears to be counter-productive when there is no occluder to complete, and the visibility gate does not suppress the completion path in that situation.

**Occluded region.** Partitioning by region (Table 8) shows that on the 395 images whose occluder covers the eye or forehead region, the adopted model gains +2.19 points over the baseline. Lower-face and side occlusion give smaller gains of +0.68 and +0.33 points, respectively. Accuracy itself is lowest on multiple or large-area occlusion (87.13%), which is the case in which the visible region carries least expressive evidence, and this is also where the baseline is weakest (84.80%).

**What the errors look like.** The errors are strongly directional. The four most frequent confusions are Sad, Happy, Disgust and Surprise all predicted as Neutral, and 21 of the 41 all-seed failures are predicted Neutral. Per class, accuracy is 96.61% on Happy and 93.72% on Neutral but 74.47% on Disgust and 80.00% on Fear. The pattern is consistent with the model defaulting to the least-marked class when occlusion removes the evidence that distinguishes an expression, rather than failing at random, and it accounts for most of the remaining error on this subset.

## 5. Discussion

**Why text, and why CLIP.** The role of text is more limited than an oracle interpretation would suggest. At inference time, the ground-truth emotion is unknown, and the decoder receives the entire bank of class-level text embeddings rather than the embedding of the correct class alone. The ablations of Section 4.3 clarify how this conditioning contributes. Removing the text pathway reduces accuracy from 91.42% to 90.60%, while replacing the prompt embeddings with seven learnable vectors containing no linguistic information yields 90.92%. Together with the component ablation, in which most of the observed text-pathway gain is associated with the alignment objective, these results indicate that the benefit cannot be attributed solely to linguistic class semantics. We therefore interpret the class-level text bank as shared conditioning memory rather than as a semantic oracle that identifies the target emotion.

The reconstruction-target ablation leads to a similarly narrow conclusion. The adopted frozen CLIP image-token target gives the highest mean accuracy (91.42%), followed by the evaluated pixel-space target (91.14%), while removing the reconstruction loss gives 90.64%. These results support the adopted reconstruction target within the evaluated configurations, but do not establish a general superiority of feature-space reconstruction over pixel-space reconstruction.

**Why the frozen backbone matters.** TeCoRe reaches its accuracy while training only ≈27 M parameters and freezing the CLIP backbone entirely, with no landmark detector, no segmentation network and no backbone fine-tuning. This contrasts with methods that fine-tune their full visual backbone or rely on additional facial priors. A practical consequence is that a single frozen CLIP encoder can be shared across tasks, with only the task-specific completion and classification modules trained. This reduces the trainable parameter count and per-task storage, although the frozen ViT-L backbone itself remains computationally substantial and we do not claim lightweight inference.

**Scope of the state-of-the-art comparison.** Among the methods compared under the standard RAN protocol, TeCoRe achieves the highest accuracy on Occlusion-RAF-DB, on both RAF-DB pose subsets, and on both FERPlus pose subsets. On the FERPlus and AffectNet occlusion subsets, however, TeCoRe is competitive but not leading, trailing FERMixNet by 1.2 percentage points and AMP-Net by 1.9 percentage points, respectively. We therefore characterise the contribution as a favourable trade-off between occlusion robustness and training cost rather than as uniform superiority. Moreover, because the frozen CLIP backbone is stronger and larger than most conventional FER backbones, the headline comparison should be interpreted together with the backbone-matched results of Section 4.2.

**Is the gain merely backbone scale?** Because TeCoRe uses CLIP ViT-L/14@336 in its main configuration, we additionally evaluate the same completion-and-text modules with different frozen CLIP backbones and input resolutions. Table 9 reports positive mean gains over the corresponding frozen linear baseline in all three evaluated settings: +1.63 points for ViT-L/14@336, +0.41 for ViT-L/14@224, and +0.91 for ViT-B/16@224. The largest improvement is observed in the main ViT-L/14@336 configuration, but the positive gains at 224 px show that the module effect is not restricted to that particular input resolution or backbone configuration.

At the same time, these three settings do not support attributing the variation in gain solely to backbone strength or solely to input resolution. In particular, at the same 224 px resolution, the measured gain is larger for ViT-B/16 than for ViT-L/14. We therefore interpret this control conservatively: the proposed modules provide positive mean improvements across the tested frozen CLIP configurations, while the magnitude of the improvement depends on the backbone and resolution setting. The headline accuracy of Table 9 should consequently be understood as the combination of a strong frozen backbone and the additional gain from the proposed modules, rather than as evidence that the modules alone account for the advantage over conventional FER baselines.

**Sensing implications.** The intended payoff is in sensing. A driver wearing a mask, or a user whose face is partly covered by a hand or a headset, presents precisely the occluded input on which conventional FER degrades. Restoring the expressive content of the hidden region—rather than discarding it—is what allows a camera in these settings to remain informative.

### Limitations

Several limitations should be stated plainly.

**Synthetic training occlusion.** Training occlusion is synthetic (rectangular, randomly filled), whose distribution differs from real semantic occlusion such as masks or hands—a criticism made explicitly by prior work. Our evaluation subsets, however, contain *real* occlusions, so the setting is a synthetic-to-real transfer that is tested against genuine occlusion rather than against more synthetic occlusion.

**Backbone scale and lightweight deployment.** As quantified in Section 5 (Table 9), TeCoRe uses a larger backbone than most baselines, and absolute cross-method comparisons should therefore be interpreted with this difference in mind. The backbone control shows positive mean gains across all three evaluated frozen CLIP configurations, although the magnitude of the gain varies with backbone and input resolution. Whether comparable gains can be retained with substantially smaller or compressed encoders remains to be investigated.

**Single-label targets.** We train and evaluate on majority-vote labels on all three datasets, discarding the annotator distributions that FERPlus and AffectNet provide. Expressions that are genuinely ambiguous or mixed are therefore forced toward a single class by both the classification and the alignment term, and we have not measured what a soft-label objective would change.

**Within-dataset evaluation only.** Training and testing are performed within the same corpus throughout, following the standard RAN protocol. We therefore do not characterise behaviour under dataset-level domain shift, where occluder statistics, demographic composition and annotation conventions differ at once. Because the backbone is frozen, the completion decoder and the visibility gate are the only components fitted to a given corpus, and whether their benefit persists across corpora—for instance training on RAF-DB and testing directly on Occlusion-AffectNet—is not answered by our experiments.

## 6. Conclusions

We presented TeCoRe, a text-conditioned completion-and-recognition framework for occlusion-robust FER. Its distinguishing idea is to restore occluded patch tokens inside the joint vision–language embedding space of a frozen CLIP encoder, using class-level textual emotion embeddings as shared conditioning memory and as an alignment target. At inference time, the decoder receives the complete class-level text bank rather than the embedding of the ground-truth emotion. The backbone is frozen entirely and only ≈27 M parameters are trained, with no landmarks, no segmentation priors and no low-rank adaptation.

On the standard RAN occlusion and pose subsets, TeCoRe achieves the highest accuracy in our comparison on Occlusion-RAF-DB, on both RAF-DB pose subsets, and on both FERPlus pose subsets. On the FERPlus and AffectNet occlusion subsets, its accuracy is competitive but not leading. Under a backbone-matched comparison on Occlusion-RAF-DB, TeCoRe reaches 91.42% while training 27.1 M parameters, compared with 90.96% for CEPrompt with 277.9 M trainable parameters and 90.64% for CLIPER with 304.4 M. The backbone-generality control further shows positive mean gains from the proposed modules across all three evaluated frozen CLIP configurations, with the largest gain observed in the main ViT-L/14@336 setting. These results position TeCoRe as a parameter-efficient approach for adapting a strong frozen vision–language representation to occlusion-robust FER, rather than as a universally superior alternative across all backbone and dataset settings.

Future work includes training on more realistic occlusion distributions, evaluating cross-dataset and zero-shot transfer, and investigating whether the completion mechanism can retain its benefit with smaller or compressed vision–language encoders. It would also be useful to explore lightweight geometric facial cues that do not require an external landmark or segmentation model, and to conduct a more detailed token-level analysis of cross-attention, reconstructed patch representations, and visibility-gate responses. Finally, soft-label supervision and extensions to dynamic, video-based FER remain promising directions.

## Figures and Tables

**Table 1:** Overall accuracy (%) on the standard RAN occlusion and pose subsets. “–” means the paper does not report that setting. Best per column in bold. Competitor backbones differ from ours and are listed for fairness (see text). The TeCoRe row is the mean ± standard deviation over three seeds fixed in advance (42/43/44), each trained on a 90% split with its epoch selected on a held-out validation split and each test subset evaluated once (Section 3.11). Competitor rows are as published.

		RAF-DB			AffectNet			FERPlus		
Method	Backbone	Occ.	P ≥ 30	P ≥ 45	Occ.	P ≥ 30	P ≥ 45	Occ.	P ≥ 30	P ≥ 45
RAN [1]	ResNet-18	82.72	86.74	85.20	58.50	53.90	53.19	83.63	82.23	80.40
EfficientFace [2]	ResNet-50	83.24	88.13	86.92	59.88	57.36	56.87	–	–	–
MA-Net [18]	ResNet-18	83.65	87.89	87.99	59.59	57.51	57.48	–	–	–
VTFF [3]	Res18 + ViT-B	83.95	87.97	88.35	62.98	60.61	61.00	84.79	88.29	87.20
CRS-CONT [19]	ResNet-18	84.63	88.37	87.99	–	–	–	–	–	–
AMP-Net [20]	ResNet-34	85.28	89.75	89.25	**64.27**	61.37	61.16	85.44	88.52	87.57
MRAN [4]	ResNet-18	86.94	90.38	89.78	63.54	**61.42**	**62.05**	–	–	–
FG-AGR [5]	ResNet-18	88.15	91.02	90.50	64.24	61.26	61.15	85.79	88.38	87.52
FERMixNet [6]	ResNet-18	86.67	89.01	87.63	62.66	60.49	61.14	**85.95**	88.55	86.73
**TeCoRe (ours)**	ViT-L/14 (frozen)	$91.42_{\pm 0.62}$	$91.39_{\pm 0.31}$	$91.10_{\pm 0.08}$	$62.42_{\pm 1.02}$	$60.01_{\pm 0.37}$	$59.76_{\pm 0.51}$	$84.79_{\pm 0.84}$	$88.46_{\pm 0.30}$	$88.05_{\pm 0.32}$

**Table 2:** CLIP-based FER on Occlusion-RAF-DB under the same CLIP ViT-L/14@336 backbone setting. CLIPER and CEPrompt were adapted from their original CLIP ViT-B/16@224 configurations to CLIP ViT-L/14@336 for a backbone-matched comparison with TeCoRe. All rows were retrained under the protocol of Section 3.11 and averaged over the same three pre-fixed seeds. “Trainable” is the peak number of parameters passed to the optimiser; CLIPER and CEPrompt fine-tune the CLIP image encoder, whereas TeCoRe keeps it frozen. Δ denotes the accuracy difference between TeCoRe and each comparison method. Boldface identifies the proposed method, the highest accuracy, and the lowest trainable parameter count.

Method	Backbone	Trainable	Occ.-RAF-DB	Δ vs. Ours
		(M)	(%)	(pp)
CLIPER [11]	CLIP ViT-L/14@336	304.4	$90.64_{\pm 0.93}$	+0.77
CEPrompt [12]	CLIP ViT-L/14@336	277.9	$90.96_{\pm 0.17}$	+0.45
**TeCoRe (ours)**	CLIP ViT-L/14@336	27.1	$91.42_{\pm 0.62}$	—

**Table 3:** Additive ablation on Occlusion-RAF-DB (mean ± standard deviation over three pre-fixed seeds). Each row adds one mechanism to the row above; Δ is measured against the frozen-backbone baseline (R0). Boldface identifies the full model, the highest accuracy, and the largest gain over R0.

Configuration	Occ.-RAF-DB (%)	Δ occ. vs. R0
R0: frozen CLIP + linear head (baseline)	89.78±0.22	—
R1: + gated completion decoder (image-feature recon., no text)	90.60±0.40	+0.82
R2: + text pathway (memory + align.) = **full TeCoRe**	91.42±0.62	+1.63

**Table 4:** Control experiment on Occlusion-RAF-DB examining whether the benefit of the text pathway depends on linguistic content (mean ± standard deviation over three pre-fixed seeds). Differences are measured relative to the prompt-based model. Boldface denotes the highest mean accuracy.

Configuration	Occ.-RAF-DB (%)	Prompt Advantage
Prompt-based text pathway (full TeCoRe)	91.42±0.62	—
Seven learnable random vectors (no linguistic content)	90.92±0.36	+0.50
Text pathway removed (R1)	90.60±0.40	+0.82

**Table 5:** Component-level ablation on Occlusion-RAF-DB (mean ± standard deviation over the same three pre-fixed seeds). Each panel varies one design axis with the rest of TeCoRe fixed; Δ is the full model minus that row on Occlusion-RAF-DB. Boldface marks the highest mean accuracy within each panel.

Variant	Occ.-RAF-DB (%)	Δ occ.
*(a) Role of the textual guidance*		
Text as cross-attention memory + alignment target (full)	91.42±0.62	—
Text as alignment target only (no memory)	91.33±0.42	+0.09
Text as memory only (no alignment loss)	90.69±0.23	+0.73
No text (completion decoder only, =R1)	90.60±0.40	+0.82
*(b) Reconstruction target of $L_{\mathrm{rec}}$*		
Frozen CLIP image token—feature space (adopted)	91.42±0.62	—
Pixel space (linear patch head, patch-normalised MSE)	91.14±0.62	+0.27
Frozen CLIP text embedding—feature space	91.01±0.62	+0.41
No reconstruction loss	90.64±0.28	+0.77
*(c) Gated-blend design*		
Soft visibility gate (adopted)	91.42±0.62	—
No gate (reconstruct all tokens)	90.19±0.39	+1.23
Hard, thresholded gate	89.74±0.28	+1.68

**Table 6:** Parameter budget of TeCoRe. Only the completion head is trained; the CLIP backbone is frozen. The text encoder is evaluated once before training to pre-compute the fixed class-level emotion embeddings and is not part of the inference graph. Boldface highlights the total trainable-parameter count.

Module	Parameters	Status
Text-conditioned completion decoder	25.2 M	trained
Visibility gate + projections + classifier	1.86 M	trained
**Total trainable**	27.06 **M**	trained
CLIP ViT-L/14@336 vision encoder + visual projection	304.3 M	frozen
CLIP text encoder (pre-computation only)	123.7 M	frozen
Inference-time footprint (vision path only)	≈331 M	—

**Table 7:** Accuracy (%) on Occlusion-RAF-DB partitioned by occluder type. Every image of the 734-image subset was assigned exactly one type from the image alone, without reference to any model output. R0 is the frozen backbone with a linear head, R1 adds the completion decoder without text, and R2 is the adopted model; all entries are means over the same three seeds, ± one standard deviation. Δ is the accuracy gain of R2 over R0 in percentage points. Categories below the rule contain fewer than 50 images and are reported for completeness only. Boldface marks the overall (All) row and the corresponding full-model accuracy.

Occluder Type	*N*	R0 (Linear)	R1 (No Text)	R2 (TeCoRe)	Δ
Eyeglasses	193	91.02±0.30	91.71±1.04	93.26±0.90	+2.25
Hand	101	89.11±0.99	90.10±1.71	90.76±0.57	+1.65
Hair	95	91.23±1.22	92.28±1.61	92.63±1.05	+1.40
Headwear	78	90.60±0.74	91.45±0.74	92.74±0.74	+2.14
Object	72	87.96±0.80	90.28±2.41	90.74±2.89	+2.78
Sunglasses	52	88.46±0.00	90.38±1.92	91.03±2.22	+2.56
Crop/out-of-frame	39	89.74±0.00	82.05±4.44	83.76±1.48	−5.98
Overlay	37	84.68±1.56	90.99±1.56	90.09±1.56	+5.41
No occluder visible	34	88.24±0.00	90.20±1.70	90.20±1.70	+1.96
Shadow/lighting	20	91.67±2.89	90.00±0.00	91.67±2.89	0.00
Other person/body	8	87.50±0.00	87.50±0.00	83.33±7.22	−4.17
Mask/cloth	5	100.0±0.00	93.33±11.6	93.33±11.6	−6.67
**All**	734	89.78	90.60	91.42	+1.63

**Table 8:** Accuracy (%) on Occlusion-RAF-DB partitioned by the facial region the occluder covers, using the same images, protocol, seeds and notation as Table 7. This partition is orthogonal to the one by occluder type: each image appears exactly once in each table. Boldface marks the overall (All) row and the corresponding full-model accuracy.

Occluded Region	*N*	R0 (Linear)	R1 (No Text)	R2 (TeCoRe)	Δ
Upper (eyes/forehead)	395	90.38±0.25	91.65±0.67	92.57±1.25	+2.19
Lower (mouth/chin)	148	90.09±1.03	89.64±0.39	90.77±1.70	+0.68
Side/half	100	90.33±0.58	89.67±1.15	90.67±0.58	+0.33
Multiple/large	57	84.80±1.01	87.72±1.75	87.13±2.03	+2.34
None visible	34	88.24±0.00	90.20±1.70	90.20±1.70	+1.96
**All**	734	89.78	90.60	91.42	+1.63

**Table 9:** Backbone-generality control on Occlusion-RAF-DB under the leak-free protocol of Section 3.11 (mean ± standard deviation over the same three pre-fixed seeds). The completion-and-text modules (Δ=R2−R0) are added on top of a frozen linear-probe baseline (R0), with every other setting held fixed. The gain is positive in all three settings.

Backbone	Res.	Patches	R0 (Linear)	R2 (Full)	Δ
CLIP ViT-L/14	336	576	89.78±0.22	91.42±0.62	+1.63
CLIP ViT-L/14	224	256	88.78±0.42	89.19±0.65	+0.41
CLIP ViT-B/16	224	196	84.79±0.06	85.69±0.51	+0.91

## Data Availability

This study analyses only publicly available benchmark datasets—RAF-DB, FERPlus, AffectNet and AffectNet+—obtained from their original providers under the respective licences and access agreements; RAF-DB and AffectNet require a signed academic-use request to their authors. No new datasets were generated in this study.
